# Differentiation Epitopes Define Hematopoietic Stem Cells and Change with Cell Cycle Passage

**DOI:** 10.1007/s12015-022-10374-4

**Published:** 2022-05-03

**Authors:** Laura R. Goldberg, Mark S. Dooner, Elaine Papa, Mandy Pereira, Michael Del Tatto, Yan Cheng, Sicheng Wen, Peter J. Quesenberry

**Affiliations:** Division of Hematology/Oncology, Rhode Island Hospital/Brown University, 1 Hoppin St Coro West Building suite 5.01, Providence, RI 02903 USA

**Keywords:** Hematopoietic stem cells, Lineage positive cells, Stem cell purification, Cell cycle, Differentiation

## Abstract

Hematopoietic stem cells express differentiation markers B220 and Gr1 and are proliferative. We have shown that the expression of these entities changes with cell cycle passage. Overall, we conclude that primitive hematopoietic stem cells alter their differentiation potential with cell cycle progression.

**Graphical Abstract:**

Murine derived long-term hematopoietic stem cells (LT-HSC) are cycling and thus always changing phenotype. Here we show that over one half of marrow LT-HSC are in the population expressing differentiation epitopes and that B220 and Gr-1 positive populations are replete with LT-HSC after a single FACS separation but if subjected to a second separation these cells no longer contain LT-HSC. However, with second separated cells there is a population appearing that is B220 negative and replete with cycling c-Kit, Sca-1 CD150 positive LT-HSC. There is a 3–4 h interval between the first and second B220 or GR-1 FACS separation during which the stem cells continue to cycle. Thus, the LT-HSC have lost B220 or GR-1 expression as the cells progress through cell cycle, although they have maintained the c-kit, Sca-1 and CD150 stem cells markers over this time interval. These data indicate that cycling stem cells express differentiation epitopes and alter their differentiation potential with cell cycle passage.

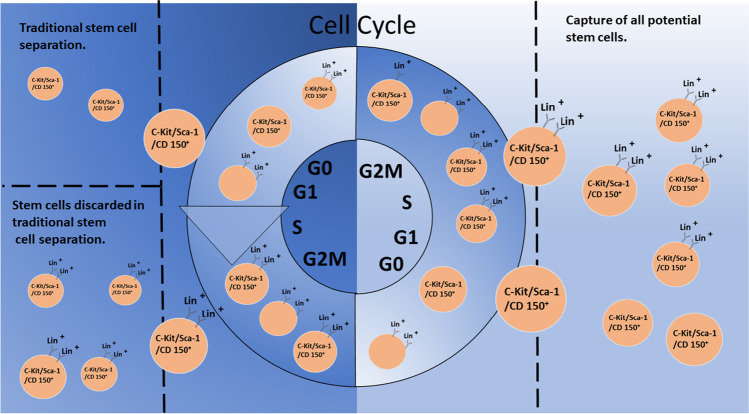

## Introduction

Hematopoietic stem cells (HSCs) have the tremendous capacity to self-renew and give rise to all the differentiated blood cells. The leading model of hematopoiesis holds that HSCs are predominantly quiescent and their production of increasingly lineage-restricted progeny occurs in a hierarchical fashion [1, 2]. Numerous studies characterizing stem cell function within sub-populations of cells purified from marrow have yielded several signature immunophenotypes of potent HSCs. Virtually all studies to date on HSCs have incorporated a lineage depletion step, with further isolation based on the presence or absence of particular cell surface epitopes [3, 4]. Such strategies yield increasingly homogenous smaller subsets of marrow cells with tremendous self-renewal and repopulation capacity. The concept of quiescence is based on studies of these purified HSCs [5–8], and nearly all our knowledge of murine HSC biology is based on studies of these highly purified quiescent HSCs.

However, our data indicate that there is a large population of cycling stem cells within un-separated murine whole bone marrow (WBM), that these cells give rise to multi-lineage peripheral blood chimerism in both primary and secondary transplant, and that this population of cycling HSCs is being preferentially discarded during conventional stem cell purification [9]. These data suggest that WBM contains a much larger, more heterogeneous population of HSCs than would be encompassed within the currently accepted models.

In these studies, we demonstrate that there is a population of cycling cells within the primary sorted Lin + population with long-term multi-lineage marrow repopulation capacity. This population has a fluctuating differentiation potential as manifest by cycle related changes of B220 and GR-1 and is positive for classic stem cell markers.

## Materials and Methods

### Mice

Congenic B6.SJL-Ptprc^a^Pepc^b^/BoyJ and C57BL/6 J male mice, age 6–8 weeks (Jackson Laboratory) were housed in accordance with the National Research Council’s Guide for the Care and Use of Laboratory Animals. All studies were in compliance with the National Institutes of Health recommendations and our Institutional Animal Care and Use Committee (Animal Welfare Assurance # A3922-01).

### Marrow Cell Preparation

C57BL/6 J (CD45.2) or B6.SJL (CD45.1) mice, referred to as CD45.2 and CD45.1, respectively, were euthanized using isoflurane inhalation followed by cervical dislocation. Femora, tibiae and iliac crests were flushed using a 22.5 gauge needle in 1X phosphate-buffered saline + 5% heat-inactivated fetal calf serum + 1% penicillin–streptomycin (PBS-HIFCS). For purified HSC isolation, a low-density mononuclear fraction was isolated from WBM using Optiprep™ (Accurate Chemical, Westbury NY) per manufacturer’s instructions. Cells were incubated with lineage-specific antibodies against B220, CD8a, CD4, CD11b, TER119, and GR1 (BD Biosciences, San Jose CA) (0.1–0.5ug/1 × 10^6^ cells) for 15 min on ice. Antibody-bound lineage positive (Lin^+^) cells were removed with Dynabeads® (Life Technologies, Carlsbad CA) per manufacturer’s protocol. The remaining lineage negative (Lin^−^) cells were recovered and incubated with allophycocyanin (APC)-conjugated c-Kit (BD Biosciences), Pacific Blue (PB)-conjugated Sca-1, phycoerythrin (PE)-conjugated CD150, FITC-conjugated CD41, FITC-conjugated CD48 (BioLegend, San Diego CA) and FITC-conjugated rat polyclonal IgG (eBioscience, San Diego CA) [0.25 μg/10^6^ cells]. Cells were washed, stained with 1μg/mL propidium iodide (PI) (Sigma-Aldrich, St Louis MO) and sorted using fluorescence-activated cell sorting (FACS) to obtain the PI-/PB + /APC + /PE + /FITC- population (viable Lin-/C-Kit + /Sca-1 + /CD150 + /CD41-/CD48- cells). All antibodies used in these studies have been tittered.

For Lin^+^ cell isolation, WBM was stained with APC- or PE-conjugated antibodies against B220, CD4, CD8a, CD11b, TER119, GR1, CD3 and CD5 (30 min on ice) and fluorescently labeled cells were isolated by FACS. These primary sorted Lin + cells, representing 96–98% of whole bone marrow cells, were double sorted with the same gating schema, yielding two populations: 1) cells persistently positive on the second sort (double sorted Lin + cells) and 2) cells falling into the negative gate on the second sort-referred to as the “double sort discard”. For stem cell marker positive sub-populations, cells were incubated with fluorescently tagged antibodies directed against c-Kit, Sca-1, and CD150 and populations positive or negative for these markers were isolated by FACS.

### Tritiated Thymidine (^3^H-thymidine) Suicide

CD45.1-derived donor cells were incubated with 200 μCi of ^3^H-thymidine (Perkin Elmer, Waltham MA) per 5 × 10^6^ cells/1 ml volume or with 10 mM unlabeled thymidine (Sigma-Aldrich) at 37˚C/5% C0_2_ for 30 min [9]. Cells were washed with PBS/10% fetal calf serum/100 µg/ml unlabeled thymidine and re-suspended in PBS. Un-manipulated cells were a negative control. Mixtures of 1 × 10^5^ or 1 × 10^6^ experimental CD45.1 cells (mixed with 300,000 or 1 × 10^6^ un-manipulated competitor CD45.2 WBM cells, respectively) were injected via tail vein into lethally irradiated CD45.2 mice.

### Morphology Studies

Marrow cell sub-populations were isolated as detailed above. Cells were prepared for microscopy using a Cytospin™ Centrifuge (Shandon, Cambridge UK) per manufacturer’s protocol. 150,000 cells/slide were prepared and Wright-Giemsa staining was performed. At least 150 cells/slide × 2 slides/prep were counted and characterized as non-proliferating granulocytes, proliferating granulocytes, lymphocytes, and erythrocytes based on morphological appearance.

### Immunophenotype Studies

WBM was labeled with PB-conjugated anti-B220, PE-conjugated anti-CD4, PE-conjugated anti-CD8, BUV395 conjugated anti-TER 119, APC-conjugated anti-GR1 (BD Biosciences), and Alexa Fluor 488 conjugated anti-CD11b (BioLegend) antibodies. For the B220 double sort discard population, B220 positive (B220 +) cells were isolated by FACS. This primary sorted B220 + population was centrifuged at 1300 rpm for 10 min at 4 °C, the pellet re-suspended in PBS and subjected to reanalysis using the same gating schema as the primary sort. Two populations of cells on the re-analysis, those persistently positive for the B220 and those negative for the B220 (the B220 double sort discard population), were analyzed for the presence or absence of the remaining Lin + markers by flow cytometry. GR1 + cells isolated on primary sort were similarly subjected to double sorting and Lin + marker analysis as above. For stem cell marker analysis, WBM was incubated with APC-conjugated antibodies against either GR1 or B220 (BD Biosciences), and FITC-labeled anti-c-Kit (BD Biosciences), PB-labeled anti- Sca-1 (BioLegend) and PE-labeled anti-CD150 (BioLegend) antibodies. Primary sorted GR1 + cells or B220 + cells were then re-analyzed and percent stem cell marker positive cells in the different cellular populations was determined by flow cytometry.

### Transplantation

CD45.2 recipient mice received 950 cGy irradiation (Gammacell® 40 Exactor, 137Cesium source irradiator, Best Theratronics Ltd. Ontario Canada) in two split fractions (475 cGy/fraction at 0.94–0.96 Gy/min) three hours apart. Experimental CD45.1 cells mixed with un-manipulated CD45.2-derived WBM cells were injected via tail vein 2 h later (24 h later for tritiated thymidine experiments). Numbers of cells injected per mouse are detailed in the figures. Peripheral blood (via tail vein bleeds) and marrow chimerism and lineage specificity were determined by flow cytometry (BD LSR II™ flow cytometer, BD Biosciences) using fluorescently tagged antibodies against CD45.1, CD45.2, B220, and CD3 (BD Biosciences) for lymphoid percentage of donor and host peripheral blood cells, and against CD45.1, CD45.2, CD11b, and GR-1 (BD Biosciences) for myeloid percentage of donor and host peripheral blood cells. Labeling was done in PBS + 0.5%FBS for 30 min at 22 °C. Pharm Lyse™ was used per manufacturer’s protocol (BD Biosciences). The percent engrafted donor CD45.1 cells was calculated as [the number of CD45.1 positive cells/ (the number of CD45.1 positive cells + CD45.2 positive cells)] × 100).

### Statistics

Non-parametric Wilcoxon rank-sum tests and Independent Samples t Tests were used to test for significant differences in chimerism between experimental groups in the transplant experiments. Pair-wise comparisons were made and the level of statistical significance was set at 0.05. All p-values are two-sided. For limiting dilution studies, L-Calc™ software (STEMCELL Technologies, Vancouver BC) was utilized to determine the frequency of stem cells within each population, scoring any mouse with < 1% donor chimerism at 6 months post-transplant as negative for donor engraftment.

Data Sharing: Please contact Laura Goldberg for datasets and protocols not available online.

## Results

### Stem Cell Potential Within the Primary Sorted Lineage Positive Marrow Cells

**Fig. 1:**
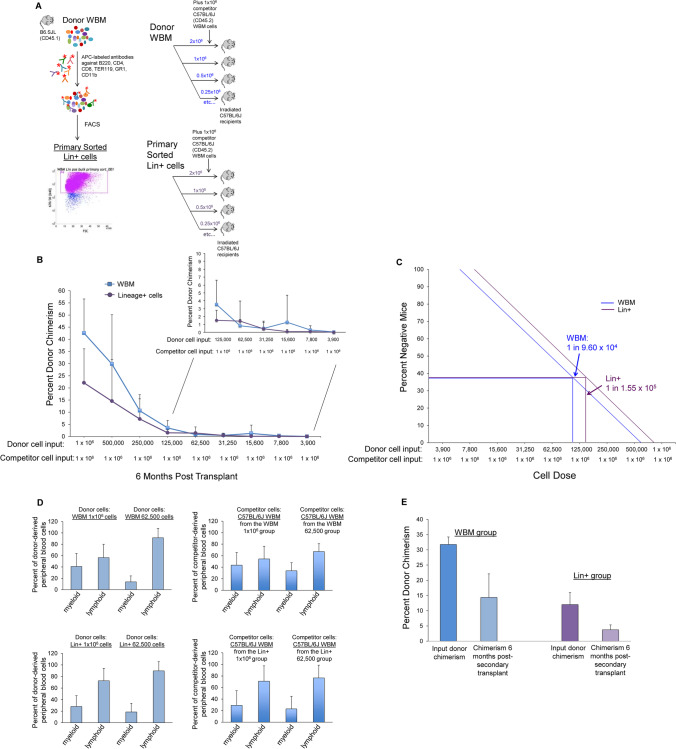
Stem cell potential within the primary sorted lineage positive marrow cell population. **(A)** Schematic of limiting dilution competitive bone marrow transplantation assay. Primary sorted Lin + cells represented 96–98% of viable WBM cells. **(B)** Engraftment of serial dilutions of primary sorted Lin + cells or WBM from CD45.1 mice when infused with 1 × 10^6^ WBM cells from CD45.2 mice into lethally irradiated CD45.2 mice at 6 months post-transplant. Data are represented as average percent donor chimerism ± SD (for donor input 1 × 10^6^ cells, *n* = 24 mice pooled from 6 independent experiments, for donor input 5 × 10^5^ and 2 × 10^5^ cells, *n* = 8 mice/dilution group pooled from 2 independent experiments, for the remining dilutions, *n* = 11–24 mice/dilution pooled from 2 independent experiments). *p < 0.05 by Wilcoxon rank sum analysis. **(C)** Data as shown in Fig. 1B were analyzed according to the Poisson distribution (L-Calc™ software, STEMCELL Technologies, Vancouver BC) to quantify the frequency of HSCs within WBM. Positive engraftment was defined as ≥ 1% donor chimerism in peripheral blood. **(D)** Lineage analysis of peripheral blood derived from donor (left panels) and competitor (right panels) cells at 6 months post-transplant for two representative serial dilutions. Data are represented as average percent of donor-derived or competitor-derived cells ± SD (*n* = 8–12 mice/dilution, data pooled from 2 independent experiments). **(E)** Marrow cells harvested at 6 months post-transplant from mice initially competitively transplanted with 1 × 10^6^ CD45.2 competitor WBM cells and either 1 × 10^6^ WBM cells or 1 × 10^6^ Lin + CD45.1 cells were serially transplanted into lethally irradiated CD45.2 mice (5 × 10^6^ cells injected per mouse). Donor chimerism, from the primary transplant from the WBM group and the Lin + group was 30–34% and 9–15%, respectively. Bars represent average percent chimerism ± SD at six months post-transplant (5–8 mice/group pooled from 2 independent experiments)

Limiting dilution studies of un-separated WBM and primary sorted Lin + marrow cells were performed. WBM and Lin + cells from donor CD45.1 were transplanted in progressively decreasing doses into lethally irradiated CD45.2 with 1 × 10^6^ competitor WBM cells derived from CD45.2 mice (Fig. 1A). Donor WBM achieved statistically significant higher levels of engraftment when compared to donor Lin + cells when doses of 1 × 10^6^, 125,000, and 15,600 donor cells were infused, while no statistically significant differences were seen at the other dilutions (Fig. 1B). Using limiting dilution competitive repopulation analysis according to the Poisson distribution to quantify the number of HSCs within WBM, with positive engraftment defined as $\ge$ 1% donor chimerism, we estimated that the frequency of competitive repopulating units was 1 in 9.6 × 10^4^ stem cells within WBM and 1 in 1.55 × 10^5^ stem cells within the Lin + population (Fig. 1C). Donor chimerism in peripheral blood was multi-lineage (Fig. 1D). These data suggest that the primary sorted Lin + population contained more than half the number of stem cells present in total unseparated WBM. Importantly, there was continued long-term engraftment capacity in the Lin + fraction in serial transplantation (Fig. 1E) although to a lesser extent than within WBM. The engraftment in secondary transplantation assays remained multi-lineage (data not shown).

**Fig. 2:**
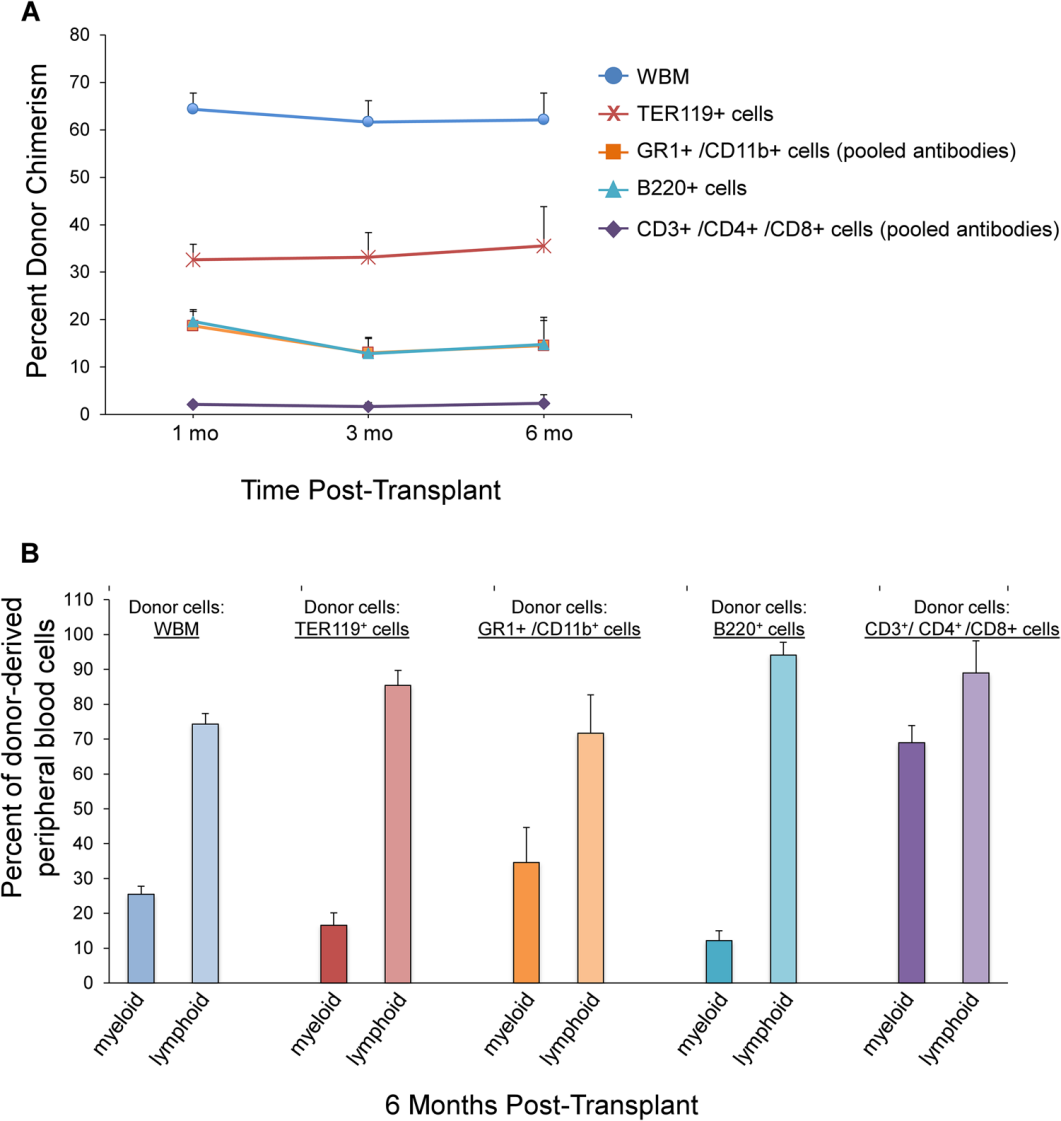
Stem cell potential in primary sorted Lineage positive subpopulations. **(A)** Engraftment of primary sorted Lin + subpopulations. 1 × 10^6^ CD45.1 donor-derived WBM, erythroid (Ter119^+^), B-lymphoid (B220^+^), 2 × 10^6^ myeloid (GR1^+^ and CD11b^+^ pooled antibodies), or 70,000 T-lymphoid (CD3^+^, CD4^+^ and CD8^+^ pooled antibodies) Lin + subsets were infused with 3 × 10^5^ CD45.2 WBM cells into lethally irradiated CD45.2 mice and peripheral blood chimerism was determined at 1, 3 and 6 months post-transplant. Data are represented as average % donor chimerism ± SEM (*n* = 8 mice/group). **(B)** Multi-lineage analysis of donor-derived peripheral blood cells at 6 months post-transplant for each donor Lin + subpopulation

Stem cell potential in primary sorted Lineage positive subpopulations. We evaluated the engraftment capacity within specific lineage positive sub-populations. Myeloid, erythroid, T-lymphoid, and B-lymphoid subpopulations isolated from CD45.1 WBM by FACS were competitively transplanted into lethally irradiated CD45.2 mice. Mice received 3 × 10^5^ un-manipulated CD45.2 WBM cells plus either: 1 × 10^6^ un-manipulated WBM, 2 × 10^6^ GR1 + and/or CD11b + cells, 1 × 10 ^6^ Ter119 cells, 70,000 CD3 + and/or CD4 + and/or CD8 + cells, or 1 × 10^6^ B220 + cells. Although disparate competition, every primary sorted individual sub-population contained long-term engraftment capacity at 6 months post-transplant, with average % donor chimerism ± SEM of 14.5% ± 5.3%, 35.5% ± 8.3%, 2.3% ± 1.8% and 14.7% ± 5.7% in the myeloid, erythroid, T-lymphoid, and B-lymphoid groups, respectively (Fig. 2A). Contribution to peripheral blood chimerism by each primary sorted Lin + sub-population was multi-lineage (Fig. 2B). Primary sorts were between 96–97.5% pure. These data indicate that there are stem cells within every primary sorted Lin + compartment with long-term multi-lineage repopulation capacity.

### Double Cell Sorting of Selected Lineage Positive Populations

**Fig. 3:**
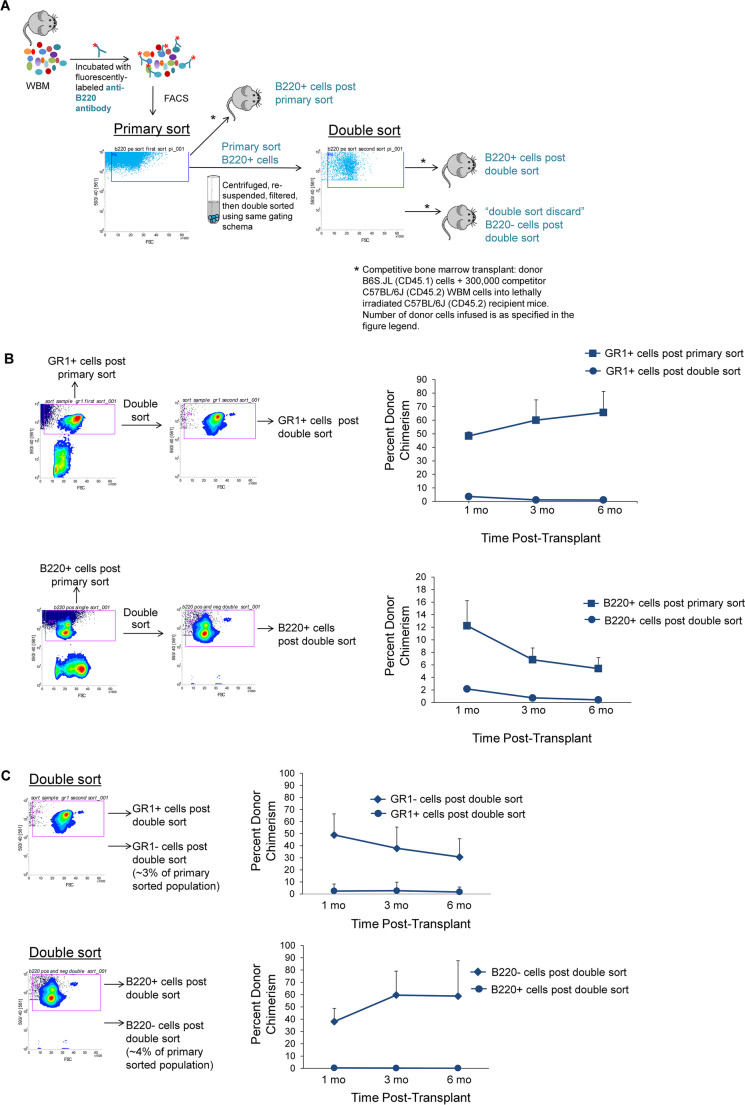
Double cell sorting of selected lineage positive populations. **(A)** Schematic of methods to test stem cell capacity within the Lin + subpopulations post primary and double sort. **(B)** Isolation (left panels) and engraftment (right panels) of donor-derived primary and double sorted GR1 + cells (top panels) and B220 + cells (bottom panels). 2 × 10^6^ GR1 + primary or double sorted cells (top panels) and 1 × 10^6^ B220 + primary or double sorted cells (bottom panels) from CD45.1 mice with 3 × 10^5^ competitor WBM cells from CD45.2 mice were infused into lethally irradiated CD45.2 mice. Data are represented as average percent donor chimerism ± SEM in peripheral blood post-transplant. (*n* = 4 mice/ GR1 group, *p = 0.006 by Independent Samples t Test, *n* = 8mice/B220 group pooled from 2 independent experiments, *p < 0.03 by Wilcoxon rank sum analysis). **(C)** GR1 + double-sorted cells and those cells that became negative for GR1 post double sort were isolated from donor CD45.1 mice (top left panel). 1 × 10^5^ donor GR1 + cells post double sort or GR1- cells post double sort cells plus 3 × 10^5^ competitor WBM cells from CD45.2 mice were infused into lethally irradiated CD45.2. Data are represented as average percent donor chimerism ± SD in peripheral blood post-transplant (*n* = 8 mice/group pooled from 2 independent experiments, *p < 0.002 by Wilcoxon rank sum analysis) (top right panel). Bottom panels show isolation (bottom left panel) and engraftment (bottom right panel) of 1 × 10^5^ donor B220 + cells post double sort or 1 × 10^5^ donor B220- cells post double sort cells plus 3 × 10^5^ competitor WBM cells. Data are represented as average percent donor chimerism ± SD (*n* = 8 mice/group pooled from 2 independent experiments, *p < 0.002 by Wilcoxon rank sum analysis)

**Fig. 4:**
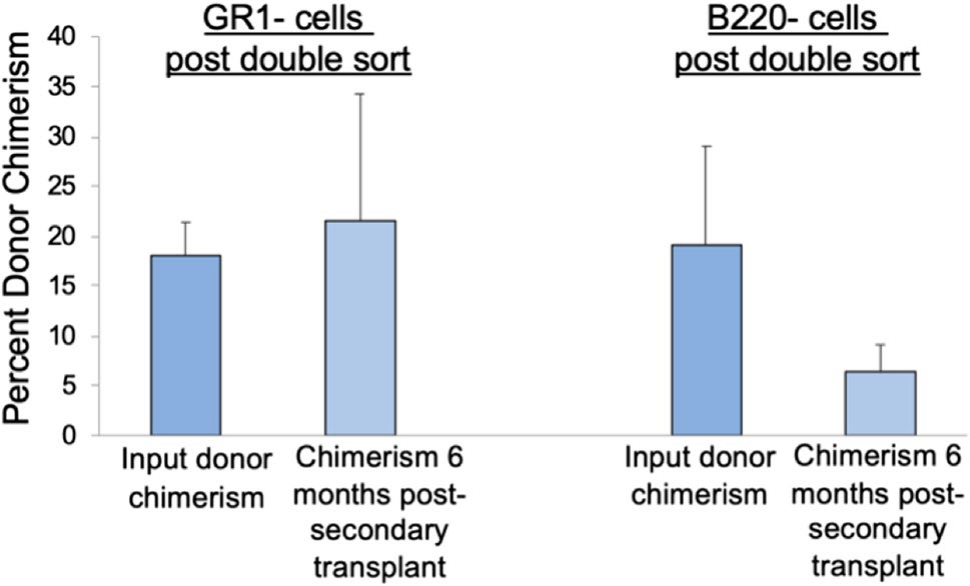
Stem cell potential in serial transplantation within the GR1- and B220- cells post double sort. Marrow cells harvested at 6 months post-transplant from mice initially competitively transplanted with 3 × 10^5^ CD45.2 competitor WBM cells and either 1 × 10^5^ GR1- cells post double sort or 1 × 10^5^ B220- cells post double sort (double sort discard populations) were serially transplanted into lethally irradiated CD45.2 mice (5 × 10^6^ cells injected per mouse). Bars represent average percent donor chimerism ± SD at six months post-transplant (7 mice/group pooled from 2 independent experiments)

We next subjected B220 and GR1 lineage selected cells to a second sort in order to evaluate a more highly purified population of Lin + cells, focusing on these sub-fractions, as they comprise a large percentage of the total Lin + population. Primary sorted GR1 + and B220 + populations were isolated by FACS, and then double sorted, and those cells persistently positive for GR1 or B220 on the double sort were tested for stem cell capacity (Fig. 3A). The double sorted, persistently Lin + cells had largely lost the previously observed stem cell capacity (Fig. 3B). On double sorting, the majority, 94–98%, remained positive for the lineage marker, but a small population of cells was present within the negative gate. In order to track the stem cell population present within the primary sorted Lin + populations, we performed double sorting on primary sorted GR1 + cells and primary sorted B220 + cells, and then collected the following populations on double sort: 1) those cells remaining positive for the selected lineage marker and 2) the small population of cells negative for that lineage marker on the second sort (Fig. 3A). These two groups were competitively engrafted into lethally irradiated CD45.2 mice. Similar to what was described above, with the second selection of either B220 or GR1, we found that we had largely lost the observed stem cell capacity in the double sorted, persistently lineage marked cells. However, there was significant engraftment within those cells negative for only the single Lin + marker post double sort (average % donor chimerism 31% ± 15% and 59% ± 29% at 6 months post-transplant for GR1 and B220 double sort discard populations respectively; 1 × 10^5 ^donor cells + 3 × 10^5 ^competitor WBM cells, *n* = 8 mice/group; *p < 0.002 by Wilcoxon rank sum analysis) (Fig. 3C) and this engraftment potential persisted in secondary transplantation (Fig. 4).

**Fig. 5:**
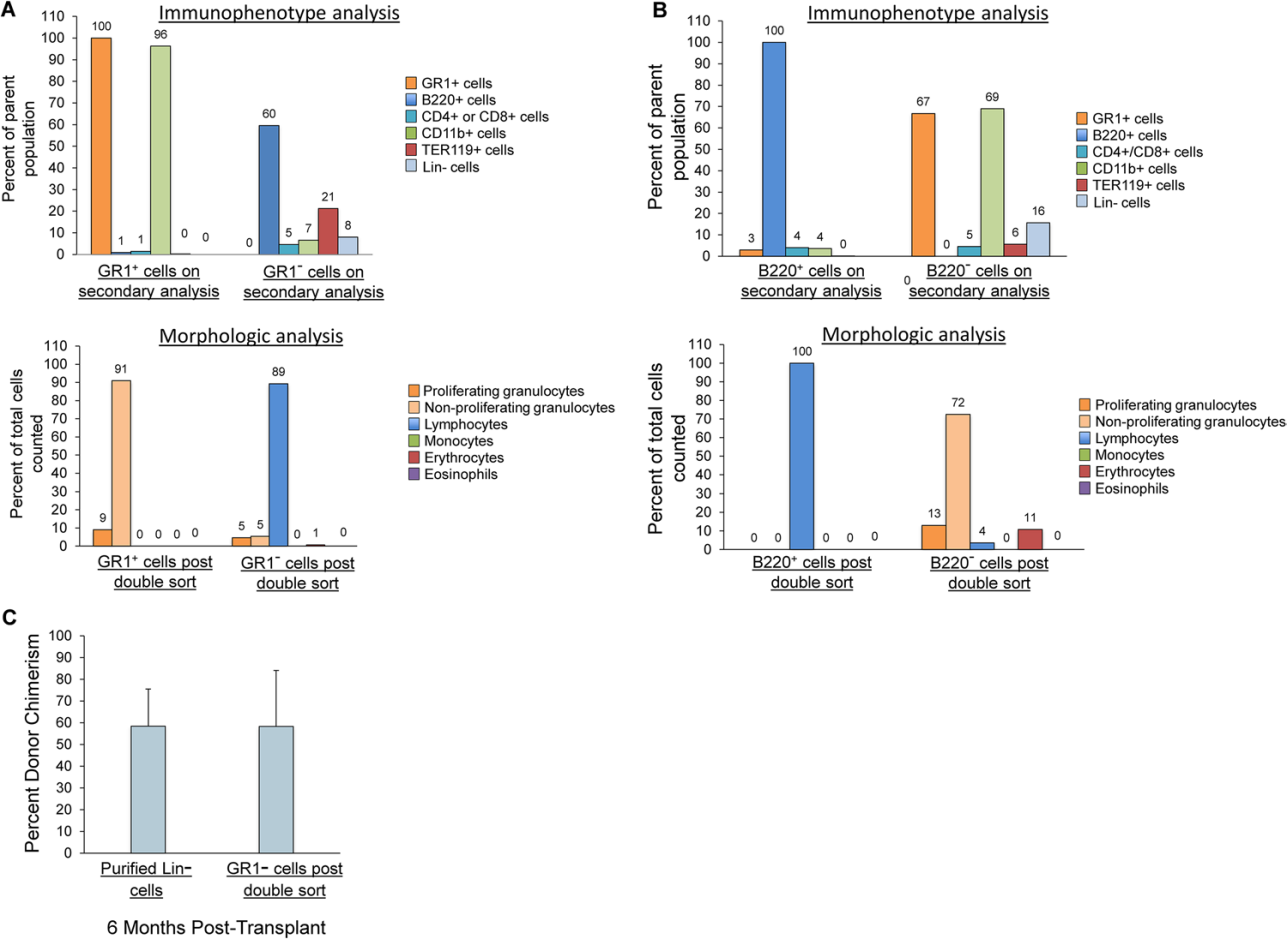
Phenotypic assessment of GR1 and B220 double sort discard populations. **(A)** Cells positive for GR1 on the primary sort, but negative for GR1 on double sort were assessed for the other Lin + markers by flow cytometry (top panel) or by morphology as visualized by microscopy (50x)(bottom panel). Each bar represents the percent of total cells positive for each lineage marker tested. **(B)** Cells positive for B220 on the primary sort, but negative for B220 on double sort were assessed for the other Lin + markers by flow cytometry) or by morphology as visualized by microscopy (50x)(bottom panel). Each bar represents the percent of total cells positive for each lineage marker tested. **(C)** Engraftment capacity of GR1- cells on double sort compared to a purified Lin- population. 1 × 10^5^ Lin- cells or 1 × 10^5^ GR1- cells on double sort and 3 × 10^5^ WBM from CD45.2 mice were injected into lethally irradiated CD45.2 mice. Bars represent average percent donor chimerism ± SD in peripheral blood at 6 months post-transplant (*n* = 3–4 mice/group, *p* = 0.99 by Independent Samples t Test)

We evaluated the morphology and immunophenotype of the double-sorted populations, including the cells initially isolated within the lineage marker positive population on primary sort but negative for the lineage marker post double sort. Despite the high engraftment capacity, the double sort cells, although negative for the single lineage marker used for the initial primary sort, contained cells positive for all the remaining lineage markers (top panels, Figs. 5A and 5B, respectively). Similarly, the B220 double sort discard population contained primarily granulocytes and erythroid cells (bottom panels, Fig. 5A and 5B, respectively). This was as expected based on the sorting schema leading to their isolation. Morphologic assessment yielded results parallel to the immunophenotypic data, with the GR1 double sort discard population containing few granulocytes but numerous lymphocytes and erythrocytes. Similarly, the B220 double sort discard population contained primarily granulocytes and erythroid cells (bottom panels, Fig. 5A and B, respectively).

The high engraftment capacity of cells isolated based solely on being negative for a single Lin + marker on double sort, despite the heterogeneous composition of the population, prompted us to directly compare their engraftment capacity to that of purified Lineage negative (Lin-) cells. We tested the GR1 double sort discard population. Despite the heterogeneity of Lin + cells within this population, based on immunophenotype and morphologic assessment, there was no statistically significant difference in average percent donor chimerism at 6 months post-transplant between purified Lin- cells and the population of GR1 positive cells that became negative for GR1 on double sort) (Fig. 5C).

**Fig. 6:**
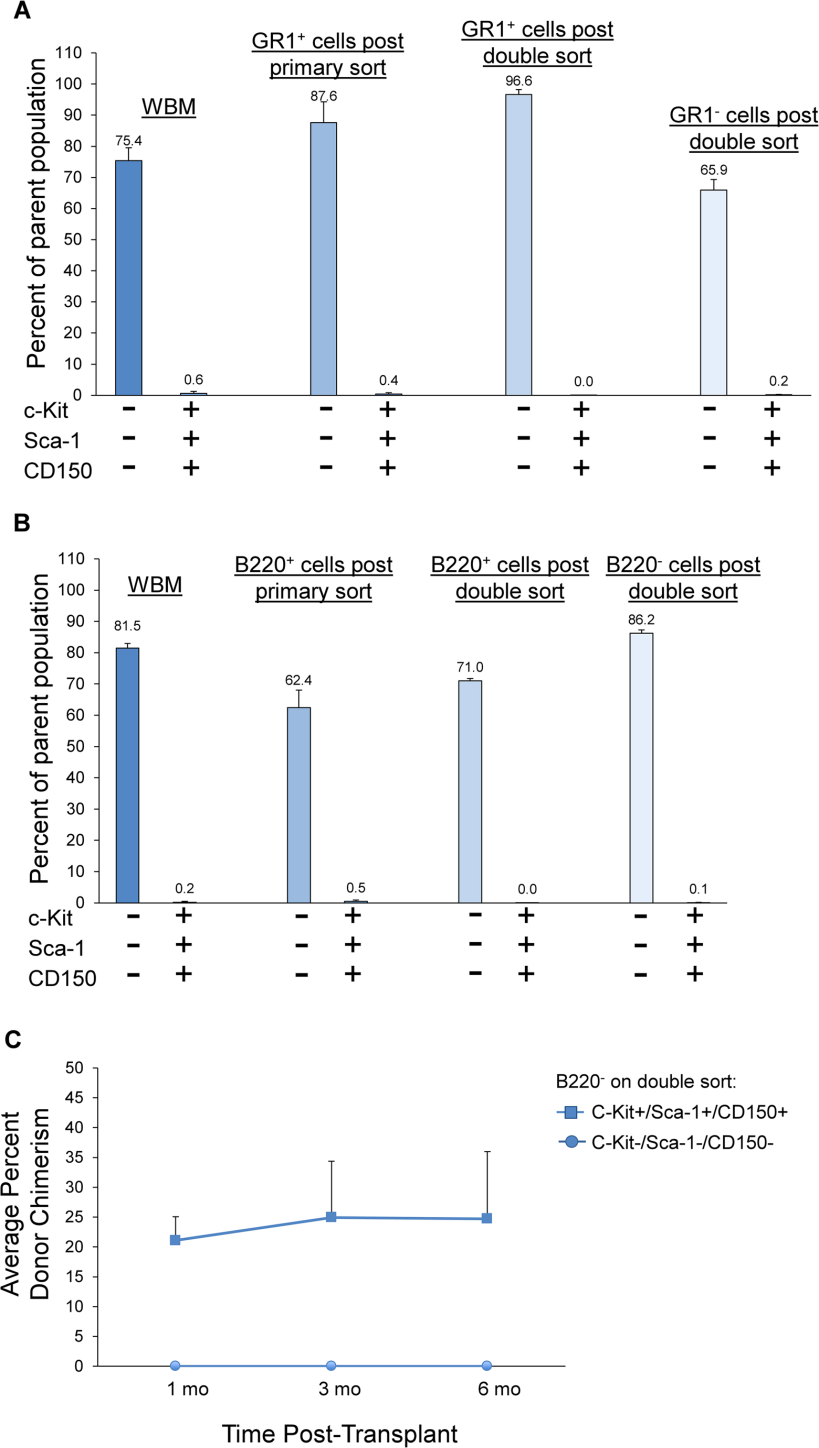
Stem cell marker expression on the Lin + and double sort discard populations. **(A)** Percent of cells within WBM, the GR1 + primary and double sorted populations and the GR1 double sort discard population that co-express c-Kit, Sca-1, and CD150 as determined by flow cytometry. Bars represent average % of population ± SD from 2 independent experiments for each population. **(B)** Percent of cells within WBM, the B220 + primary and double-sorted populations and the B220- cells on double sort that co-express c-Kit, Sca-1, and CD150. Bars represent average % of population ± SD from 2 independent experiments for each population. **(C)** Engraftment capacity within the population of B220- cells on double sort is found within the population that co-expresses c-Kit, Sca-1, and CD150. 300 B220- cells on double sort also negative for c-Kit, Sca-1, and CD150, or 300 B220- cells on double sort that were positive for all three stem cell markers were injected with 3 × 10^5^ competitor WBM cells into lethally irradiated recipient mice and peripheral blood chimerism was determined at 1, 3, and 6 months post-transplant. Data are represented as average % donor chimerism ± SEM in peripheral blood (*n* = 6–7 mice/group pooled from 2 independent experiments, *p < 0.003 by Wilcoxon rank sum analysis)

We next determined the prevalence of classical HSC markers, c-Kit, Sca-1, and CD150, on the different primary sorted Lin + subpopulations. As shown in Fig. 6, there was a very small percentage of cells positive for c-Kit, Sca-1 and CD150 within the primary sorted GR1 + cells and within the population of cells that became negative for GR1 on the double sort. No cells within the GR1 + population on double sorting were positive for all three HSC markers (Fig. 6A). Similar results were obtained for the B220 subpopulations (Fig. 6B). Utilizing the B220^−^ population on double sort, we next compared engraftment capacity between the stem cell marker-positive and stem cell marker-negative B220^−^ populations on double sort. As shown in Fig. 6C, virtually all the stem cell capacity within the B220^−^ cells on double sort was within the c-Kit + /Sca-1 + /CD150 + population.

### HSCs initially isolated from the GR1 + and B220 + primary sorted populations are cycling cells

**Fig. 7:**
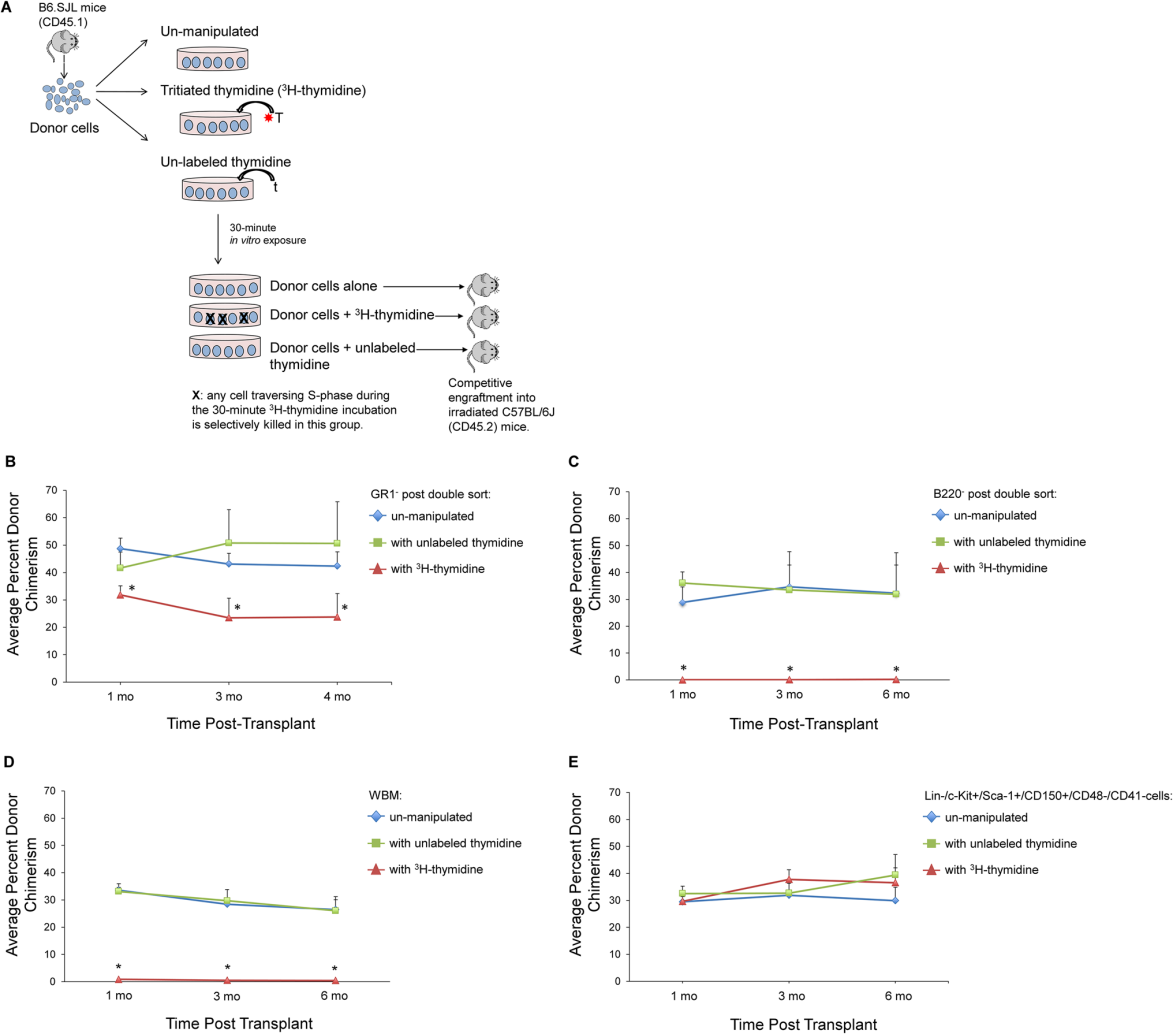
HSCs initially isolated from the GR1 + and B220 + primary sorted populations are cycling cells. (**A**) Different cell populations were incubated in the presence of tritiated thymidine (^3^H-thymidine), unlabeled thymidine, or left un-manipulated and then competitively transplanted into lethally irradiated recipient mice. Peripheral blood chimerism was determined at 1, 3, and 6 months post-transplant. (**B**) 1 × 10^5^ donor GR1 negative cells post double sort plus 3 × 10^5^ un-manipulated competitor WBM were injected into lethally irradiated recipient mice. Data are represented as average percent donor chimerism ± SEM (*n* = 4–6 mice/group pooled from 2 independent experiments). *p < 0.05 by Wilcoxon rank-sum test, ^3^H-thymidine group vs. pooled control groups (unlabeled thymidine + un-manipulated). (**C**) 1 × 10^5^ donor B220 negative cells post double sort plus 3 × 10^5^ un-manipulated competitor WBM. Data are represented as average percent donor chimerism ± SEM (*n* = 5–6 mice/group pooled from 2 independent experiments). *p < 0.008 by Wilcoxon rank-sum test, ^3^H-thymidine group vs. unlabeled thymidine and ^3^H-thymidine group vs. un-manipulated control group). (**D**) 1 × 10^6^ donor WBM and 1 × 10^6^ un-manipulated competitor WBM. Data are represented as average percent donor chimerism ± SEM (*n* = 8–12 mice/group averaged from 3 independent experiments). *p < 0.001 by Wilcoxon rank-sum test, ^3^H-thymidine group vs. unlabeled thymidine and ^3^H-thymidine group vs. un-manipulated control group). (**E**) 200 donor Lin-/c-Kit + /Sca-1 + /CD150 + /CD41-/CD48- cells and 2.5 × 10^5^ un-manipulated competitor WBM. Data are represented as average percent donor chimerism ± SEM (*n* = 12–24 mice/group averaged from 3 independent experiments; chimerism from 12–16 of these mice at the one month time-point were previously published) [9]. There was no statistically significant difference between groups at any time-point (*p* > 0.1 by Wilcoxon rank-sum test)

To further characterize the population of stem cells that are initially isolated within the Lin + population and are marker negative after double sorting, we evaluated the cell cycle status of the engrafting cells utilizing tritiated thymidine suicide (Fig. 7A). When cells from either the GR-1 or B220 double sort discard cells were incubated in the presence of tritiated thymidine and then competitively engrafted into lethally irradiated mice, there were significant reductions in engraftment by the tritiated thymidine-exposed cells, indicating that these cells had traversed S-phase (Fig. 7B, 7C). This also indicates that during the two FACS separations the stem cells continued to cycle. WBM tested in parallel showed a significant reduction in engraftment capacity with tritiated thymidine incubation (Fig. 7D). This is in contrast to the classically defined purified HSCs, which are predominantly dormant. As expected, purified HSCs were not affected by tritiated thymidine, supporting their quiescence (Fig. 7E), and supporting the presence of a cycling population of HSCs preferentially lost with conventional HSC purification**.** These data indicate that the population of stem cells within the primary sorted B220 + population that become negative on double sort are stem cell marker positive, and are a cycling population of stem cells, distinct from classically defined purified HSCs including Lin-/c-Kit + /Sca-1 + /CD150 + HSCs, which are predominantly quiescent. Altogether these data suggest that B220 positive LT-HSC progress through cell cycle and lose B220 expression. This fluctuation in differentiation potential is consistent with past studies showing that synchronized LRH cells evidenced transitory megakaryocyte and granulocyte differentiation hotspot with cycle passage [10].

## Discussion

This work confirms previously published work showing that a significant population of long-term repopulating marrow stem cells is discarded with the stem cell purification and that these cells are actively proliferating [9]. Based on limiting dilution competitive repopulation assays, we found that the stem cell frequency within the primary sorted Lin + population was approximately 50% of that found in the entire unseparated WBM. These stem cells were capable of long-term multi-lineage engraftment in both primary and secondary transplantation, and their presence within the Lin + population, a population discarded in virtually all HSC studies, indicates that we are underestimating the true number and character of HSCs within marrow. We have shown that stem cell activity is present within each of the individual primary sorted Lin + subsets including the B220 + , GR-1 + and/or CD11b + , Ter119 + and CD3 and/or CD4 and/or CD8 + cellular subsets and that, for every sub-population tested, long-term engraftment was multi-lineage.

When carrying out double cell sorts to enhance “purity,” we found that the lineage positive population post second sort had lost all or most of its stem cell capacity. However, the stem cell capacity was present in the minor population negative for the single Lin + marker used in the primary sort, which we have termed the double sort discard population. Specifically, for the B220 + and GR1 + cellular subsets, the stem cells within the double sort discard were actively cycling and, for the tested B220 double sort discard, expressed the stem cell markers c-Kit, CD150 and Sca-1. On initial consideration, it may seem the simplest explanation for the presence of stem cell activity within the primary sorted Lin + population is due to classically defined Lin-/lo/progenitor/stem cell marker positive cells contaminating the primary sort either by chance or due to a doublet discrimination error and then returning to their rightful lineage marker negative gate during the double sort. The possibility of doublets would be rare, as doublets were eliminated from all sorts using pulse width. While these settings would help to eliminate doublets from the sort, rare sorter error cannot be fully dismissed. Therefore, we acknowledge the possibility of a doublet containing a rare stem cell. However, it is worth noting, the stem cell activity within both the primary sorted Lin + population [9] and within the double sort discard populations was due to cycling stem cells. In addition, the stem cells within the primary sorted Lin + population were able to contribute to long-term multi-lineage engraftment in serial transplantation. However, this engraftment was less than that achieved by unfractionated WBM and less than reported for immunophenotypically-defined HSCs. Therefore, given their cell cycle transit phenotype coupled with their slightly diminished ability to repopulate marrow in serial transplantation, they appear distinct from classically defined purified Lin-/stem cell marker + cells, which are predominantly quiescent and with robust repopulation capacity in serial transplantation. The fact that they can contribute to long-term multi-lineage repopulation in both primary and secondary transplantation indicates they are not merely conventionally defined progenitors. Thus, they appear to be distinct both from cycling progenitors and from classically defined purified Lin-/stem cell marker + cells, indicating the stem cell activity in the primary Lin + subsets is not likely due to simple contamination by Lin- progenitor cells or conventionally defined Lin-/stem cell marker + HSCs.

We have developed a continuum model of hematopoiesis in which the stem cell phenotype is continually changing on a cell cycle related basis, with fluctuations in self-renewal potential and lineage fate linked to cell cycle transit [11–13]. The work presented here indicates that c-Kit, Sca-1 and CD150 are likely reliable stem cell epitopes and may define the true hematopoietic stem cell population, but that a significant portion of this population is actively cycling and may have fluctuating expression of lineage specific epitopes and therefore be at risk of discard during lineage depletion. We propose that proliferating stem cells with typical stem cell markers are lost in the initial lineage depletion and that their differentiation potential varies with cell cycle transit. There is abundant evidence for fluctuation of different genes and cell surface determinants with cell cycle passage, activation state and developmental stage [14–21]. Our previous studies with highly synchronized purified lineage negative, rhodamine low, Hoechst low murine hematopoietic stem cells have indicated the presence of differentiation ‘hotspots’ in early and mid-S phase for megakaryocytes and granulocytes, respectively [10], suggesting lineage predilection sites at different points in cell cycle. Studies have shown a connection between cell cycle and lineage fate choices in human embryonic cells [22]. Similarly, investigators have shown a clear link between cell cycle regulation and megakaryocyte versus erythroid lineage fate determination in megakaryocyte-erythroid progenitors [23] and have defined key links between cell cycle progression and erythroid differentiation [24]. We posit that differentiation epitopes may be expressed on the cell surface of HSCs at different points in cycle and their loss may simply reflect cycle progression of the stem cells during the time of double sorting. In this model for example, the initial stem cell with B220 expression may evolve into a stem cell with expression of an alternate marker over time. These markers may well determine the susceptibility of the stem cells to specific differentiation signals and influence lineage specification. This is our present working hypothesis.

The work presented here indicates that there is much more molecular and functional heterogeneity within the HSC pool than would be predicted by studies focused on only the small sub-populations of highly purified HSCs within marrow. This is in agreement with an emerging body of literature suggesting that highly purified, classically defined HSCs may not fully represent the entire stem cell population within marrow. There have been numerous studies indicating more heterogeneity within the well-accepted hierarchy than previously thought [25–30]. Studies have shown that granulocyte-monocyte progenitors retain lymphoid potential and early thymic progenitors retain B-cell and myeloid potential [31, 32]. Early lymphoid-biased progenitors were found to have myeloid potential, and elegant gene expression profiling studies found that these multi-lymphoid progenitors retained a relatively active HSC transcription program [33, 34]. Although classically considered to mark more differentiated T cells, there have been reports of CD4 + cells with repopulation capacity and repopulation capacity has also been found in MAC-1 + populations [35, 36]. Furthermore, studies on mRNA expression in purified stem cells have indicated that primitive stem cells express a wide variety of putative regulators and markers. These can vary with stage of cell cycle [15–17, 21]. In addition, analyses of multipotent hematopoietic stem and progenitor cell transcriptional programs by single cell RT-PCR indicated several lineage-affiliated gene expression programs were primed prior to final commitment to a single lineage [37]. This lineage priming model is further supported by elegant studies by Ye et al., in which, utilizing a Cre-Lox approach to fate map cells expressing lysozyme, a myelomonocytic marker, they found that lysozyme expression was not only in myeloid committed cells but also in long-term repopulating HSCs [38]. Finally, although distinct from a transplantation setting, in steady-state hematopoiesis, Sun et al. showed that it is not the conventionally defined HSCs that were the drivers of hematopoiesis but rather long-lived progenitors [39] suggesting that these progenitors, as well as the cell population described in this paper, may be a component of a larger population of cells that includes the classical hematopoietic stem cell. Additional studies have shown that megakaryocyte progenitors may represent the major fate of the classical long-term hematopoietic stem cells [40, 41]. Other work has described different stem/progenitor cells underlying steady state hematopoiesis and a hierarchy of uni- and oligo-lineage clones within multi-potent progenitor populations [42]. It is conceivable that the afore-mentioned continuum model would include these steady state clones.

In summary, although the rare purified stem cell is clearly a cell with tremendous self-renewal and differentiative potential, numerous studies, in combination with our work presented here indicate the existence of a population of stem cells within the Lin + marrow fraction that is discarded during most HSC isolation strategies which classically focus on only the Lin^−/lo^/stem cell marker^+^ HSCs. These data underscore the existence of a more extensive, heterogeneous and dynamic HSC pool within marrow important for hematopoiesis in classic transplantation conditions. We hypothesize that the cycling stem cells within the primary sorted Lin + population described in this work are a major population of hematopoietic stem cells capable of repopulating the marrow, characterized by a relatively stable set of stem cell markers (c-Kit, Sca-1 and CD150), but with a continuous fluctuation of cell surface markers and differentiation potential. Our future work will be aimed at determining how cell cycle related fluctuations in phenotype influence key HSC functions of self-renewal and lineage fate choices within this population of HSCs and whether differentiation potential of LT-HSC extends well beyond the hematopoietic realm.

## Data Availability

Please contact Laura Goldberg for datasets and protocols not available online.
